# Evaluation of Adolescents for Polycystic Ovary Syndrome in an Urban Population

**DOI:** 10.4274/jcrpe.v1i4.50

**Published:** 2010-12-08

**Authors:** Sarabeth Broder Fingert, Bina Shah, Marion Kessler, Melissa Pawelczak, Raphael David

**Affiliations:** 1 Department of Pediatric Endocrinology, New York University School of Medicine, New York, NY; +1-212-562-3793sbf241@med.nyu.eduNew York University School of Medicine 435 East 30th St, Rm 301 New York, NY 10016

**Keywords:** Polycystic ovary syndrome, PCOS, adolescent, morbidity

## Abstract

**Objective**: To assess the quality of diagnostic work-up received by patients with “possible” polycystic ovary syndrome (PCOS).

**Design**: A retrospective chart review.

**Setting**: A hospital based Pediatric Clinic in New York City.

**Patients**: Sixty female patients aged 13-19 years, with a primary ICD-9 diagnosis of ovarian dysfunction (256), menstrual irregularity (626), or hirsutism (704.1) were randomly selected for evaluation. In addition, 18 patients who were assigned the same ICD-9 codes at the Pediatric Endocrine Clinic were assessed.

**Main Outcome**: Rates of assessment for diagnostic criteria of PCOS and selected co-morbidities.

**Results**: Twenty-five percent (15/60) of the patients were evaluated for PCOS according to the Rotterdam Criteria, and only 2 were evaluated for common co-morbidities associated with PCOS. Of the 28 patients who presented with two or more signs of PCOS (menstrual irregularity plus either obesity, hirsutism and/or acne), 15 were evaluated for PCOS (54%), but only 7% were assessed for common co-morbidities.

All patients referred to the Pediatric Endocrine Clinic received appropriate evaluation for PCOS. In addition, 89% of the study group underwent further assessment for selected complications of PCOS.

**Conclusions**: Patients presenting to an inner-city pediatric clinic with “possible” PCOS often do not receive a complete diagnostic evaluation. In addition, those evaluated for PCOS are often not adequately screened for the known health consequences associated with this condition. These findings suggest that PCOS is under evaluated and possibly under diagnosed in this pediatric population, which raises serious concerns regarding the potential for major longterm public health consequences.

**Conflict of interest:**None declared.

## INTRODUCTION

Polycystic ovary syndrome (PCOS) is a common disorder with an estimated prevalence of 5-10% in women of reproductive age.^1^ The prevalence has been increasing in the adolescent population.^2^ In more than 40% of cases, PCOS is associated with obesity, ^3^ as well as impaired glucose tolerance, type 2 diabetes, and the metabolic syndrome.^4^ Furthermore, in adult female patients it is associated with an increased risk of endometrial cancer and is the most common identifiable cause of female infertility. ^1, 2, 3, 4, 5, 6^ While the pathophysiology of PCOS remains unclear, insulin resistance has been implicated as a major causative factor.^7^ In addition to this, several genes have been associated with this syndrome.^8^

There is an increasing awareness of PCOS among the adolescent population^9^ along with an increase in diagnosis and an increased incidence of established co-morbidities such as obesity and type 2 diabetes.^2, 10^ Even so, diagnosis in this population can often be elusive.^11^ Accurate diagnosis of PCOS is of critical importance to public health, given the chronic nature of the disorder and its association with multiple health consequences.^11^

PCOS has many presentations and multiple definitions have been suggested for this disorder. ^8^ Also, it may mimic other disorders such as congenital adrenal hyperplasia and androgen secreting neoplasms.^4^ Given the variability of signs, symptoms, biochemical and radiologic features in a given individual, diagnosis of PCOS can be easily missed. Moreover, even when PCOS is correctly diagnosed screening for other metabolic constellations may not be consistently carried out. Therefore, we analyzed the quality of the evaluation process for PCOS and associated conditions in a population of adolescent girls presenting to urban Pediatric and Pediatric Endocrine clinics with the possible diagnosis of PCOS.

## METHODS

**Study Design and Patient Population**

The study was approved by an institutional review board and a retrospective chart review was performed. Medical records of patients aged 13-19 years who presented in 2005-2006 with a “possible” diagnosis of PCOS were evaluated by a single investigator (Table 1). “Possible PCOS” was defined according to the ICD-9 codes of ovarian dysfunction (256.0), menstrual irregularity (626.0) or hirsutism (704.1) either alone or in combination. ICD-9 codes of diabetes mellitus (250.0) and obesity (278.0) were also recorded due to their association with PCOS. Patients with known causes (i.e. pregnancy, malignancy or congenial adrenal hyperplasia) of these conditions were excluded. One thousand forty patients with possible PCOS were identified, and 60 were selected for analysis by simple random sampling (i.e. 60 charts were randomly selected from the 1040 charts which were manually reviewed).

**A. Criteria for the diagnosis of PCOS**

Each patient was evaluated for the quality of diagnostic work-up for PCOS according to the Rotterdam Criteria.^8^ This included evaluation for at least two of the following: menstrual irregularity, clinical or biochemical evidence of hyperandrogenism, and ultrasonographic (either transvaginal or transabdominal) features suggestive of PCOS. Patients who received at least two of these evaluations were considered adequately evaluated for PCOS.

**B. Assessment of features associated with PCOS**

Eight specific criteria selected for their diagnostic and prognostic association with PCOS were assessed. These included: menstrual irregularities, hirsutism, ovarian ultrasound, fasting glucose, fasting insulin, lipid profile (HDL, LDL and triglycerides), testosterone, and FSH to LH ratio. Patients who were tested for all eight criteria were considered fully evaluated. Those who were tested for <8 but >2 criteria were considered partially evaluated.

**Evaluation in the pediatric clinic**

Sixty patients were randomly selected and studied for the quality of the diagnostic evaluation they received for PCOS and features associated with PCOS.

**Evaluation in the subspecialty Pediatric Endocrine Clinic**

As a quality control, 18 patients who were referred and evaluated in 2005-2006 at the Pediatric Endocrine clinic of the same hospital were analyzed. These patients were assessed according to the same criteria for both diagnostic evaluation and associated findings of PCOS.

**Table 1:**
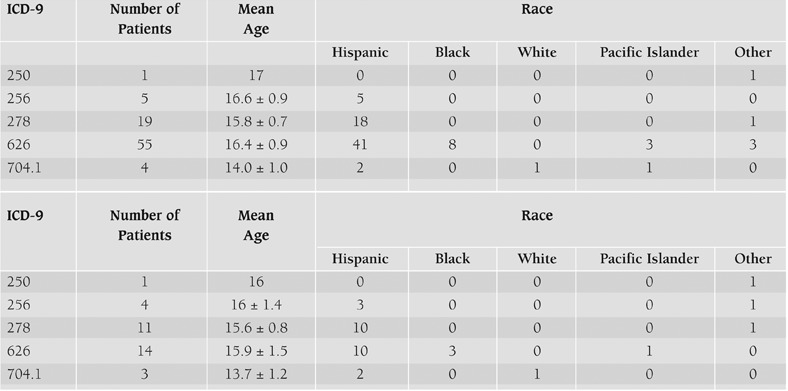
Demographics of patients according to ICD-9 code. A, General Pediatric Clinic. B, Pediatric Endocrine Clinic. ICD-9 codes: ovarian dysfunction (256), menstrual irregularity (626), hirsutism (704.1), diabetes mellitus (250) and obesity (278).

## RESULTS

**Figure 1:**
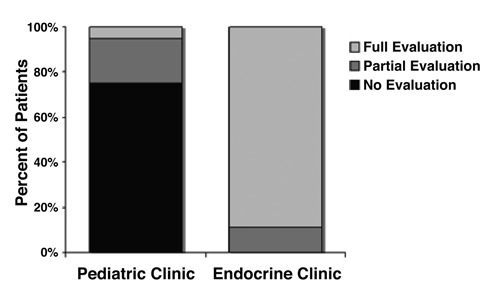
Percentage of patients (%) evaluated for PCOS among 60 patients with “possible” PCOS. NE, patients not evaluated with any of the following: testosterone levels and/or ovarian ultrasound, FSH/LH, fasting blood glucose or lipid panel; PE, patients partially evaluated with testosterone levels and/or ovarian ultrasound, but not FSH/LH, fasting blood glucose or lipid panel; FE, patients fully evaluated with testosterone levels and/or ovarian ultrasound, FSH/LH, fasting blood glucose or lipid panel.

Twenty five percent (15/60) of the patients seen in the general pediatric clinic, were considered to have received an adequate diagnostic evaluation for PCOS according to the Rotterdam Criteria (i.e. evaluation for menstrual irregularity, hyper-androgenism or ovarian ultrasound features, Table 1, Figure 1). Of the 15 patients adequately evaluated for PCOS, 6 (40%) fulfilled the Rotterdam diagnostic criteria for PCOS, yet only 3 (50%) were coded as PCOS (Table 2).

**Figure 2:**
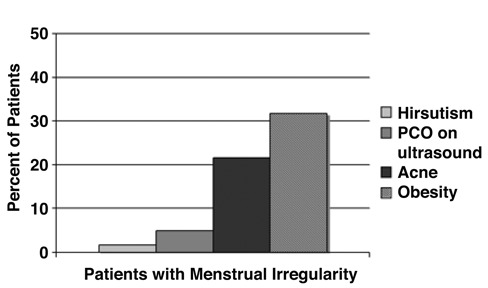
Most common additional diagnoses according to ICD-9 code in 60 patients with “possible” PCOS in General Pediatric Clinic.

**Figure 3:**
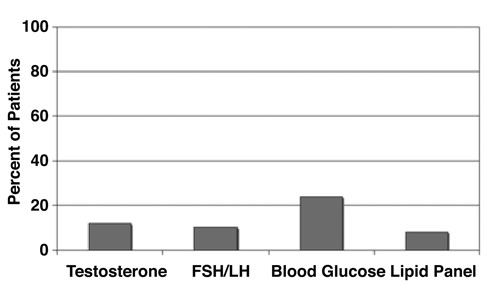
Percentage of patients (%) with “possible” PCOS in General Pediatric Clinic who received selected laboratory evaluations.

Of the initial 60 patients, 36 had other findings in addition to menstrual irregularity. Of these 36 patients with additional findings, 32% were obese, 21% had acne, and 7% were hirsute (all associated with elevated testosterone levels and PCO appearance on ultrasound, Figure 2), however only 15 were evaluated for PCOS (54%), and only 7% received evaluation addressing a selected group of findings most commonly associated with PCOS (Figure 3). Furthermore, only 2 of the 6 patients with findings diagnostic for PCOS were evaluated for selected associated medical conditions (data not shown).

Of the 1040 patients in the original population identified according to the ICD-9 diagnostic codes, 18 who were referred to the Pediatric Endocrine Clinic were included in the analysis (Table 1). All of the referred patients received adequate diagnostic evaluation for PCOS (Figure 1, Table 2). Moreover, the majority of these patients (89%) were also assessed for medical conditions known to be associated with PCOS (Figure 1).

**Table 1:**
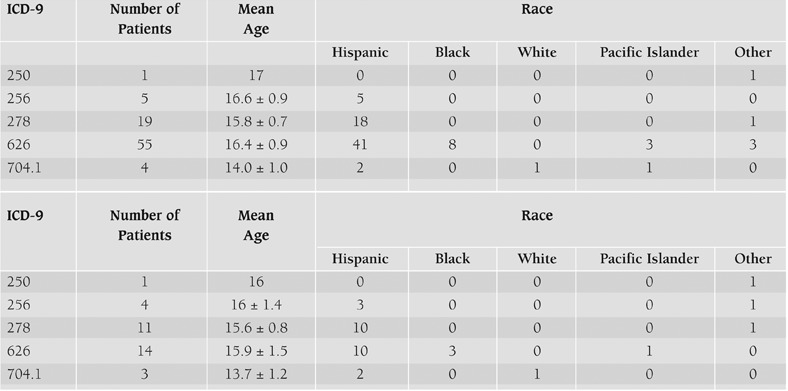
Demographics of patients according to ICD-9 code. A, General Pediatric Clinic. B, Pediatric Endocrine Clinic. ICD-9 codes: ovarian dysfunction (256), menstrual irregularity (626), hirsutism (704.1), diabetes mellitus (250) and obesity (278).

**Table 2:**
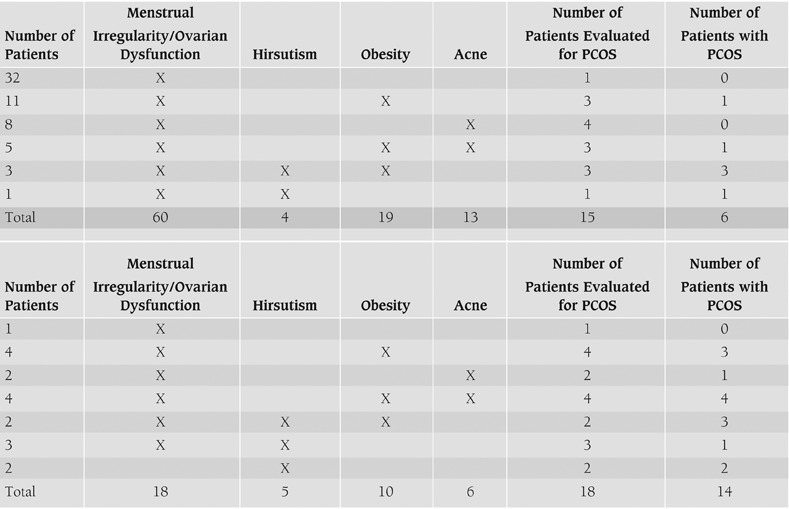
Most common ICD-9 coded diagnoses in patients with “possible” PCOS. A, General Pediatric Clinic. B, Pediatric Endocrine Clinic.

## DISCUSSION

PCOS can be a diagnostic quandary for pediatricians for many reasons. Adolescents may not always seek medical attention for oligo or amenorrhea,^12^ and if they do, they are often prescribed oral contraceptive pills (OCPs) to regulate their menses without being offered an endocrinologic evaluation. Many of these patients will remain on OCPs until the time of desired fertility, often many years later, which will mask any prolonged menstrual disturbances, as well as features of hyperandrogenism. With these difficulties in mind, we reviewed the data obtained from adolescent girls presenting with a “possible” diagnosis of PCOS in an urban hospital- based clinic. We demonstrated that these patients are not adequately evaluated for PCOS or its associated complications.

PCOS may present in the adolescent years, ^9^ but limited data are available on its prevalence or pathophysiology specifically for this age group.^13^ Despite the paucity of data, it is clear that PCOS confers a physical, emotional and economic burden on both adults and adolescents affected with this condition.4 Accurate diagnosis at a younger age may be a key to preventing many of the long-term health consequences associated with this syndrome. In our population of patients with “possible” PCOS, only 25% received a sufficient diagnostic work-up for this condition. Even patients who presented with obesity, hirsutism and/or acne (all associated with PCOS) in addition to menstrual irregularity as a subgroup, were evaluated for PCOS only 50% of the time. Of those evaluated for PCOS, nearly half had PCOS according to diagnostic criteria^8^ but were not coded or treated as such. These data indicate that nearly half of adolescents presenting with PCOS in thispopulation may be missed. On the other hand, the 25% of patients who were initially evaluated may have presented with clinically more severe symptoms, and could represent a higher proportion of actual PCOS cases. In either case, it is likely that a significant number of cases of PCOS in the adolescent population are being missed.

In addition, even when patients are assessed for PCOS in the primary care setting, they are often not evaluated for known co-morbidities. Less than 10% of patients were tested for impaired glucose tolerance or hyperlipidemia. Type 2 diabetes mellitus and metabolic syndrome have been increasing in both the adult and pediatric populations, ^15, 16^ and are strongly associated with PCOS.^17^ Therefore, a missed diagnosis of PCOS and its co-morbidities may be a missed opportunity to halt the development of these complications.

On the other hand, all patients evaluated in the Pediatric Endocrine Clinic received a full evaluation for PCOS. In a survey of pediatric endocrinologists, 67% of respondents reported that they would measure testosterone in a patient that presented with oligomenorrhea alone.^14^ Consistent with our findings, these data indicate that pediatric endocrinologists often perform a more exhaustive work-up for PCOS than general pediatricians. In addition, endocrinologists and gynecologists evaluate and manage PCOS differently.^18^ In women with suspected PCOS, endocrinologists were more likely to evaluate androgen, lipid and glucose levels, while gynecologists were more likely to order ovarian ultrasounds. It is clear that there is no consensus between pediatricians, endocrinologists, and gynecologists regarding the evaluation and management of adolescents with PCOS.

In conclusion, the present study suggests that PCOS is being underevaluated and possibly underdiagnosed in the adolescent population. PCOS is associated with many health consequences, both of immediate (poor selfesteem, acne^9^) and long-term (diabetes, heart disease, metabolic syndrome, infertility^10^) concern. Awareness of PCOS and its diagnosis must be increased among physicians caring for adolescent girls. A policy statement on the diagnosis of PCOS specifically addressing the adolescent population may be warranted for adequate diagnosis of this disorder.
